
JOURNAL INFORMATION
==============================
NLM Title Abbreviation: Campbell Syst Rev
Journal ID: Campbell Syst Rev
NLM Journal ID: 9918227275506676
Journal ID: CL2

Campbell Systematic Reviews

EISSN: 1891-1803
Publisher: SAGE Publications

ARTICLE INFORMATION
==============================
PMCID: PMC12048860
DOI: 10.1002/cl2.70046
Article ID: CL270046
Article version: 1
Subjects: Issue Information, Issue Information

Issue Information

Electronic publication date: 2025 May 3
Publication date: 2025 Jun
Volume: 21
Issue: 2
Electronic Location ID: e70046
Revised 2024 Dec 16; Received 2024 Jul 1; Accepted 2025 Jan 29
Copyright: © 2025 The Author(s). Campbell Systematic Reviews published by John Wiley & Sons Ltd on behalf of The Campbell Collaboration.
License: This is an open access article under the terms of the http://creativecommons.org/licenses/by/4.0/ License, which permits use, distribution and reproduction in any medium, provided the original work is properly cited.
License URL: https://creativecommons.org/licenses/by/4.0/

Figure count: 0
Table count: 0
Page count: 11

source-schema-version-number: 2.0
cover-date: June 2025
details-of-publishers-convertor: Converter:WILEY_ML3GV2_TO_JATSPMC version:6.5.6 mode:remove_FC converted:12.05.2025

==============================

VOLUME 21 · ISSUE 2 · JUNE 2025

### CAMPBELL SYSTEMATIC REVIEWS

Open Access

BETTER EVIDENCE FOR A BETTER WORLD

WILEY

Campbell Collaboration

### Campbell Systematic Reviews

EDITOR IN CHIEF
**Vivian Welch**
Bruyère Research Institute, Ottawa, Canada

### Aims and Scope

*Campbell Systematic Reviews* is an open access journal prepared under the editorial control of the Campbell Collaboration. The journal publishes peer reviewed systematic reviews, evidence and gap maps, and methods research papers.

Campbell has a growing list of editorial topic areas, including Ageing, Business and Management, Children & Young Persons Wellbeing, Climate Solutions, Crime and Justice, Disability, Education, International Development, Knowledge Translation and Implementation, Methods, Social Welfare, Training.

Campbell promotes positive social and economic change by enabling evidence-based policy and practice. Our vision is "Better evidence for a better world".

TECHNICAL ADVISORY GROUP

Ruth Garside, Exeter University, Truro, UK
Larry Hedges, Northwestern University, Evanston, USA
Julia Littell, Bryn Mawr College, Bryn Mawr, USA
Terri Pigott, Georgia State University, Atlanta, USA
James Thomas, University College London, London, UK
David Tovey, Co-Editor in Chief, Journal of Clinical Epidemiology and Emeritus Editor in Chief, Cochrane, UK

EDITORIAL BOARD

**Ageing**
Jo Thompson Coon, University of Exeter, Exeter, UK
Claire Goodman, University of Hertfordshire, Hatfield, UK

**Business and Management**
Eric Barends, Centre for Evidence Based Management (CEBMa), Leiden, The Netherlands
Denise Rousseau, Carnegie Mellon University, Pittsburgh, USA

**Children and Young Persons Wellbeing**
Kerry Albright, UNICEF, New York, USA
Nathan Hughes, Sheffield University, UK

**Climate Solutions**
Jan Minx, Mercator Research Institute on Global Commons and Climate Change, Berlin, Germany
Hugh Sharma Waddington, London School of Hygiene & Tropical Medicine, University of Aberdeen, UK

**Crime and Justice**
Angela Higginson, Queensland University of Technology, Brisbane, Australia
Dorothy Newbury-Birch, Teesside University, Middlesbrough, UK

**Disability**
Joann Starks, American Institutes for Research (AIR), Washington, D.C., USA
Oliver Wendt, University of Potsdam, Germany

**Education**
Roisin Corcoran, University of Texas Health Science Center at Houston, USA
Sarah Miller, Queen's University Belfast, Belfast, UK

**International Development**
Marie Gaarder, International Initiative for Impact Evaluation (3ie), London, UK
Peter Tugwell, University of Ottawa, Ottawa, Canada

**Knowledge Translation and Implementation**
Xinsheng 'Cindy' Cai, American Institutes for Research (AIR), Washington, D.C., USA
Kathleen Murphy, American Institutes for Research (AIR), Washington, D.C., USA

**Methods**
Ruth Garside, University of Exeter, UK
Gavin Stewart, University of Newcastle upon Tyne, UK

**Social Welfare**
Douglas Besharov, University of Maryland, College Park, USA

**Sexual orientation and gender identity Sub-group: Social Welfare**
Kelly Haviland, Memorial Sloan Kettering Cancer Center, New York, USA
Zack Marshall, University of Calgary, Calgary, Canada

**Substance Use Treatment and Recovery Sub-group: Social Welfare**
Emily A. Hennessy, Harvard Medical School, Boston, USA
Noel Vest, Boston University School of Public Health, USA

**Training**
Jeffrey Valentine, University of Louisville, Louisville, USA

EDITORS

**Ageing**
Jo Thompson Coon, University of Exeter, Exeter, UK

**Business and Management**
Sandra Buttgieg, University of Malta, Malta
Denise Rousseau, Carnegie Mellon University, Pittsburgh, USA

**Children and Young Persons Wellbeing and Social Welfare Groups**
Fiona Campbell, Newcastle University, UK

**Climate Solutions**
Ruth Garside, University of Exeter, Truro, UK
Denny John, M S Ramaiah University of Applied Sciences, Karnataka, India
William Lamb, Mercator Research Institute on Global Commons and Climate Change, Berlin, Germany
Jan Minx, Mercator Research Institute on Global Commons and Climate Change, Berlin, Germany

Ashrita Saran, Campbell South Asia, New Delhi, India
Jan Petzold, Ludwig-Maximilians-Universität München, Germany
Zohra Lassi, Robinson Research Institute at the University of Adelaide, Australia

**Crime and Justice**
Elizabeth Eggins, Griffith University, Brisbane, Australia
Angela Higginson, Queensland University of Technology, Brisbane, Australia
Lisa Tompson, University of Waikato, Hamilton, New Zealand

**Disability**
Brian Reichow, UConn Health, University of Connecticut, Farmington, USA

**Education**
Sarah Miller, Queen's University Belfast, Belfast, UK

**International Development**
Maren Duvendack, University of East Anglia, Norwich, UK
Shari Krishnaratne, London School of Hygiene and Tropical Medicine, UK
Elizabeth Kristjansson, University of Ottawa, Ottawa, Canada
Ashrita Saran, Campbell South Asia, New Delhi, India
Fahad Siddiqui, Duke-NUS Medical School, Singapore

Shannon Shisler, International Initiative for Impact Evaluation (3ie), Buffalo, USA
Birte Snilstveit, International Initiative for Impact Evaluation (3ie), London, UK
Elizabeth Ghogomu, University of Ottawa, Ottawa, Canada

**Knowledge Translation and Implementation**
Xinsheng 'Cindy' Cai, American Institutes for Research (AIR), Washington, D.C., USA

**Methods**
Ruth Garside, University of Exeter, UK
Gavin Stewart, University of Newcastle upon Tyne, UK

**Social Welfare**
Fiona Campbell, Newcastle University, UK
Brandy Maynard, Saint Louis University, Saint Louis, USA
Heid Nøkleby, Norwegian Institute of Public Health, Oslo, Norway

**Sexual orientation and gender identity Sub-group: Social Welfare**
Zack Marshall, University of Calgary, Calgary, Canada

**Substance Use Treatment and Recovery Sub-group: Social Welfare**
Emily A. Hennessy, Harvard Medical School, Boston, USA

### METHODS EDITORS

**Ageing**
Ruth Garside, University of Exeter, Exeter, UK
Dylan Kneale, University College London, London, UK
Kristin Liabo, University of Exeter, Exeter, UK
Morwenna Rogers, University of Exeter, Exeter, UK

**Business and Management**
Kevin D. Carlson, Virginia Tech, Blacksburg, USA
Katerina Gonzalez, Suffolk University, Boston, USA
Laura Michaelson, American Institutes for Research (AIR), Washington, D.C., USA

**Children and Young Persons Wellbeing**
Terri Pigott, Georgia State University, Atlanta, USA

**Climate Solutions**
Kristin Konnyu, University of Aberdeen, UK
Wolfgang Viechtbauer, Maastricht University, Maastricht, The Netherlands
Hugh Sharma Waddington, London School of Hygiene & Tropical Medicine, University of Aberdeen, UK

**Crime and Justice**
GJ Melendez-Torres, University of Exeter, Exeter, UK

**Disability**
Derek Rodgers, University of Iowa, Iowa City, USA

**Education**
Peter Boedecker, Baylor College of Medicine, USA
Darren Moore, University of Exeter, Exeter, UK

**International Development**
Emily A. Hennessy, Harvard Medical School, Boston, USA

**Knowledge Translation and Implementation**
Sean Grant, University of Oregon, Eugene, Oregon, USA

**Methods**
Max Callaghan, Mercator Research Institute on Global Commons and Climate Change, Germany
Belén Fernández-Castilla, National University of Distance Education, Spain
Hanan Khalil, La Trobe University, Australia
Jihyun Lee, The University of North Texas, USA
Joshua R. Polanin, American Institutes for Research (AIR), Washington, D.C., USA
Xuping Song, Lanzhou University, China

**Social Welfare**
Terri Pigott, Georgia State University, Atlanta, USA
Mikkel Helding Vembye, Danish Center for Social Science Research (VIVE), Copenhagen, Denmark

**Sexual Orientation and Gender Identity Sub-group: Social Welfare**
GJ Melendez-Torres, University of Exeter, Exeter, UK

**Substance Use Treatment and Recovery Sub-group: Social Welfare**
Robert A. Marx, San Jose State University, USA

**Social Welfare Associate Methods Editors**
Kamal Middlebrook, Georgia State University, Atlanta, USA
Joseph Morris, Georgia State University, Atlanta, USA

### INFORMATION SPECIALISTS

**Ageing**
Morwenna Rogers, University of Exeter, UK

**Business and Management**
Zahra Premji, University of Victoria, Victoria, Canada
Ryan Splenda, Carnegie Mellon University, Pittsburgh, USA

**Children and Young Persons Wellbeing**
Rocio Rodriguez Lopez, Andalusian Health Technology Assessment Agency, Fundación Progreso y Salud, Seville, Spain

**Climate Solutions**
Alison Bethel, University of Exeter, UK

**Disability**
Amy Riegelman, University of Minnesota, USA

**Education**
Patrick Labelle, University of Ottawa, Canada

**International Development**
Kirsten Elliott, University of Nottingham, Nottingham, UK
Heather MacDonald, Carleton University, Ottawa, Canada

**Knowledge Translation and Implementation**
Stephen Maher, New York University, USA

**Methods**
Sarah Young, Carnegie Mellon University, Pittsburgh, USA

**Social Welfare**
Scott Marsalis, University of Minnesota Libraries, USA
Amalia Monroe-Gulick, University of Kansas, USA
Morwenna Rogers, University of Exeter, UK

**Sexual Orientation and Gender Identity Sub-group: Social Welfare**
Scott Marsalis, University of Minnesota Libraries, USA

**Substance Use Treatment and Recovery Sub-group: Social Welfare**
Leonard Levin, Harvard Medical School, Boston, USA

### ASSOCIATE EDITORS

**Climate**
Suchi Malhotra, Campbell South Asia, Bangalore, India

**Crime and Justice**
Alexander Trinidad, University of Cologne, Cologne, Germany
Amy Nivette, Utrecht University, Utrecht, Netherlands
Kiran Sarma, University of Galway, Galway, Ireland

**Disability**
Karen McConnell, Queen's University Belfast, Belfast, UK

**Education**
Emma Dobson, Newcastle University, Newcastle, UK
Erin Early, Ulster University, Belfast, UK
Louise Gascoine, Durham University, Durham, UK
Maria Katsipataki, Durham University, Durham, UK
Leonor Rodriguez, The University of Edinburgh, Scotland, UK

**International Development**
Hannah Chirgwin, Foreign, Commonwealth and Development Office in London, UK
Thomas De Hoop, American Institutes for Research (AIR), Washington, D.C., USA
Mark Engelbert, International Initiative for Impact Evaluation (3ie), London, UK
Damian Francis, Georgia College and State University, Midgeville, USA
Laurenz Langer, South Africa Centre for Evidence, Johannesburg, South Africa
Tamara Lofti, McMaster University, Ontario, Canada
Jennifer Petkovic, Bruyère Research Institute, Ottawa, Canada
Daniel Phillips, Education Endowment Foundation, London, UK
Jennifer Stevenson, Independent consultant, London, UK
Fiona Wallace, Nutrition Opportunities Worldwide, Toronto, Canada

**Social Welfare**

**Sexual orientation and gender identity Sub-group: Social Welfare**
Kelly Haviland, Memorial Sloan Kettering Cancer Center, New York, USA

**Substance Use Treatment and Recovery Sub-group: Social Welfare**
Brandon Bergman, Harvard Medical School, Boston, USA
Charlotte Colman, Ghent University, Belgium

### EDITORIAL ADVISORY BOARD OF PEER REFEREES

Brandon Bergman, Harvard Medical School, Boston, USA
Charlotte Colman, Ghent University, Belgium
Thomas Abt, Department of Criminology and Criminal Justice, University of Maryland, USA
Cynthia Anaba, School of Public Health, Kwame Nkrumah University of Science and Technology (KNUST), Ghana
Laura Boira, Evidence Hub of Latin America and the Caribbean, Brazil, Brazil
Alisha Bruton, Center for Mental Health Innovation, Department of Psychiatry, Oregon Health & Science University (Portland, OR)
Hans Bugge, Bergsund Norwegian Institute of Public Health, Norway
Sarah Carthy, Institute of Security and Global Affairs, Leiden University, Ireland
Rodrigo Conde, Faculdade Anhanguera, Ribeirão Preto, Brazil
Andi Coombes, American Institutes for Research, USA
Nicola Deghaye, Stellenbosch University (affiliate), South Africa
Omar Dewidar, University of Toronto, Canada
Jessica Ferguson, Teesside University, UK
Nicholas Gage, WestEd
Tadesse Gebrye, Health Economics and Outcomes, Department of Health Professions, Faculty of Health and Education, Manchester Metropolitan University
Liping Guo, Lanzhou University, China
Alexa von Hagen, Individual Development and Adaptive Education (IDeA) Research Center in Frankfurt am Main, Germany
Liangying Hou, Lanzhou University, China
Laura Huey, University of Western Ontario
Jade Hutchinson, Charles University, Czech Republic
Jiri Kantor, Center of Evidence-Based Education & Arts Therapies, Palacký University Olomouc, Czech Republic
Xiuxia Li, Lanzhou University, China
Yanfei Li, Lanzhou University, China
Ed Maguire, School of Criminology and Criminal Justice, Arizona State University, USA
Suchi Kapoor Malhotra, Director Research, Campbell South Asia. Fellow, Evaluation, and Evidence Synthesis Program, Global Development Network (GDN), New Delhi
Marilia Mastrocolla de Almeida Cardoso, Health Technology Assessment Unit of the Hospital das Clinicas da Faculdade de Medicina de Botucatu Brazil
Sinead McGilloway, Maynooth University Maynooth Co., Ireland
Ashima Mohan, Campbell South Asia, India
Nick Moore, Integrity Global, UK
Harrison Nelson, Faculty of Health Sciences, Queen's University, Kingston, Canada
Veronica Osorio, Evidence Hub of Latin America and the Caribbean, Bolivia, Bolivia
Bei Pan, Lanzhou University, China
Shefton J Parker, Monash University
Erica Phillips, Department of Nutritional Sciences, University of Wisconsin - Madison, US
Miloň Potměšil, Institute of Special Education Studies, Faculty of Education, Palacky University Olomouc, Czechia
Petra Potměšilová, Dept. of Christian Education, Sts Cyril and Methodius Faculty of Theology, Palacký University Olomouc, Czechia
Mark Reed, National Audit Office
Daniel Riquelme-Uribe, Center for Management in Education DCS-FEN University of Chile, Chile
Jana Rozmarinova, Faculty of Education, Palacký University Olomouc, Czechia
Yael Shraga-Roitman, Faculty of Education, Ono Academic College, Israel
Xuping Song, Lanzhou University, China
Joseph Stabile, Independent Scholar
Susanna Larsson Tholén, SBU – Swedish Agency for Health Technology Assessment and Assessment of Social Services
Emma Tuschick, Teesside University, UK
Matteo Vergani, Deakin University, Australia
Tereza Vrbová, Institute of Health Sciences, Faculty of Medicine, Masaryk University, Czechia
Qi Wang, Mcmaster university, Canada
Xiaoqin Wang, University of Ottawa heart institute, Canada
Behzad Zamani, Tehran University of Medical Sciences, Iran
